# The Association of 3-Hydroxy-3-Methylglutaryl-CoA Reductase, Apolipoprotein E, and Solute Carrier Organic Anion Genetic Variants with Atorvastatin Response among Jordanian Patients with Type 2 Diabetes

**DOI:** 10.3390/life10100232

**Published:** 2020-10-05

**Authors:** Hussam Alhawari, Yazun Jarrar, Mohammad Ahmad AlKhatib, Hussein Alhawari, Munther Momani, Ayman Zayed, Ruba Alkamhawi, Malek Zihlif

**Affiliations:** 1 Department of Internal Medicine, Faculty of Medicine, The University of Jordan, Amman 11942, Jordan; h.hawari@ju.edu.jo (H.A.); h.alhawari@ju.edu.jo (H.A.); munthermomani1971@gmail.com (M.M.); baraaaayman@gmail.com (A.Z.); 2 Department of Pharmaceutical Science, Faculty of Pharmacy, Al-Zaytoonah University of Jordan, Amman 11733, Jordan; 3 Haemostasis and Thrombosis Laboratory, The University of Jordan, Amman 11942, Jordan; m_alkhatib2004@yahoo.com; 4 Certified Lab Technician, Jordan University Hospital, Amman 11942, Jordan; Abutabboosh@gmail.com; 5 Department of Pharmacology, Faculty of Medicine, The University of Jordan, Amman 11942, Jordan; m.zihlif@ju.edu.jo

**Keywords:** diabetes, Jordanian, SLCO1B1, atorvastatin, genotype

## Abstract

Atorvastatin is commonly used among type 2 diabetic (DM2) patients at the University of Jordan Hospital to prevent cardiovascular complication. However, we noticed that there is a wide inter-individual variation in the efficacy and toxicity of atorvastatin. This study aimed to find out the effects of major genetic variants in 3-Hydroxy-3-Methylglutaryl-CoA Reductase (HMGCR), Apolipoprotein E (APOE), and Solute Carrier Organic Anion (SLCO1B1) genes on atorvastatin response among DM2 patients. A sample of 139 DM2 patients on 20 mg of atorvastatin was included in this study. The lipid and glycemic profile and the levels of hepatic enzymes alanine aminotransferase (ALT) and aspartate transaminase were recorded before and after 3 months of atorvastatin treatment. Additionally, the genetic variants *HMGCR rs17244841,* *APOE rs7412* and *rs429357,* and *SLCO1B1 rs2306283* and *rs11045818* were genotyped using an Applied Biosystems DNA sequencing method (ABI3730×1). We found that atorvastatin reduced total cholesterol and low-density lipoprotein (LDL) more significantly (*p*-value < 0.05) in patients with wild genotype than variant alleles *APOE rs7412C > T* and *SLCO1B1 rs2306283A > G*. Furthermore, the ALT level was elevated significantly (*p*-value < 0.05) by 27% in patients with heterozygous *SLCO1B1 rs11045818* G/A genotype, while it was not elevated among wild genotype carriers. Additionally, atorvastatin reduced total cholesterol more significantly (*p*-value < 0.05) in patients with *SLCO1B1 rs2306283A* and *rs11045818G* haplotypes and increased ALT levels by 27% (*p*-value < 0.05) in patients with *SLCO1B1 rs2306283G* and *rs11045818A* haplotypes. In conclusion, it was found in this study that *APOE rs7412, SLCO1B1 rs2306283,* and *rs11045818* genotypes can be considered as potential genetic biomarkers of atorvastatin response among DM2 patients of Jordanian Arabic origin. Further clinical studies with larger sample numbers are needed to confirm these findings.

## 1. Introduction

Atorvastatin is a strong, long-acting inhibitor of 3-hydroxy glutaryl co-enzyme A reductase (HMGCR), which is the rate-limiting step in the biosynthesis of cholesterol [1]. Atorvastatin is used widely in the treatment of hypercholesterolemia [2]. In addition, the guidelines of the American Association of Diabetes recommend using statin drugs for the prevention of cardiovascular complication of diabetes mellitus (DM) [3]. Accordingly, physicians at the University of Jordan Hospital prescribe statins, especially atorvastatin, to DM2 patients. However, there is an inter-individual variation in the atorvastatin response. Some patients still have high cholesterol levels even when they are on statin treatment, and some patients suffer from statin-induced hepatotoxicity and myopathy [4]. This inter-individual variation in drug response may be due to some environmental factors, such as food and drug–drug interactions, as well as genetic factors [5]. It is reported that statin response is altered by genetic variants in genes encoding proteins involved in the pharmacodynamics and kinetics of the drugs [6].

3-hydroxy glutaryl co-enzyme A reductase (HMGCR) and apolipoprotein E (ApoE) play a role in the pharmacodynamics of statins; HMGCR is the target of statin drugs, and ApoE is a carrier of lipids in the blood [1]. Solute carrier organic anion 1B1 (SLCO1B1) transporter is responsible for the transportation of statins into hepatic cells, which is the major site of statin activity [7]. Some studies among different ethnic groups report that genetic variants in the *HMGCR*, *APOE,* and *SLCO1B1* genes influence statin response [8,9,10].

There are no reports regarding the influence of *HMGCR*, *APOE,* and *SLCO1B1* genetic variants on statin response among DM2 patients of Jordanian Arabic origin. We hypothesized that genetic variants in these genes could at least partly explain the observed inter-individual variation in statin response. Therefore, the present study aimed to investigate the effect among Jordanian DM2 patients of major genetic variants *HMGCR rs17244841, APOE rs7412* and *rs429357,* and *SLCO1B1 rs2306283* and *rs11045818* on the atorvastatin response, which is the most commonly used statin at the University of Jordan Hospital.

## 2. Materials and Methods

### 2.1. Chemicals

Isopropanol and 70% ethanol were obtained from Sigma-Aldrich (Sant Louis, MO, USA), and 100 base pair (bp) DNA ladder and PCR master mix were purchased from Promega (Madison, WI, USA). Oligo DNA primers were purchased from Integrated DNA Technologies (Coralville, IA, USA). Agarose powder was purchased from Seakem R LE agarose (Lonza, NJ, USA).

### 2.2. Patients

This study was a cross-sectional study conducted from 11/2018 to 1/2020. The study included 139 DM2 patients who were on 20 mg of atorvastatin and were regularly attending the Diabetes Clinic at Jordan University Hospital. The patients had just started atorvastatin. We chose atorvastatin among other statins because it is the most common one used in the University of Jordan Hospital. The DM2 patients continued taking 20 mg of atorvastatin daily for 3 months. Diabetic patients were excluded if (1) they were on other statin drugs, such as rosuvastatin, pravastatin, or simvastatin; (2) they had any other chronic disease, such as cardiovascular diseases; or (3) they had missing lipid or glycemic data profiles. Ethical approval was provided by the Institutional Review Board of Jordan University Hospital (number 67/2018/225). Informed consent was obtained from every patient before starting atorvastatin treatment.

According to the records of The Jordan University Hospital, an average of 250 Jordanian Arabic DM2 patients, without any other chronic diseases, were prescribed 20 mg atorvastatin last year. It was calculated that 139 sample size represents the DM2 patients on 20 mg atorvastatin therapy using the power of test 1 − β = 0.8, 5% margin of error, and 95% confidence level.

### 2.3. Data Collection

Jordan University Hospital’s computer records were used to obtain demographic data and the results of blood lipid and glucose profile analyses for total cholesterol, low-density lipoprotein (LDL), high-density lipoprotein (HDL), triglyceride (TG), and glycated hemoglobin (HbA1c %).

### 2.4. Calculation of Atorvastatin Response

The atorvastatin response was calculated as percentage (%) as follows: 

Atorvastatin response = [(Lipid, glycemic, or hepatic enzyme level after 3 months of atorvastatin treatment-Lipid, glycemic, or hepatic enzyme level before 3 months of atorvastatin treatment)/Lipid, glycemic, or hepatic enzyme level before 3 months of atorvastatin treatment] × 100

### 2.5. Blood Collection and DNA Extraction

EDTA tubes were used to collect 5 mL venous blood samples. DNA was isolated from the whole blood using a Wizard^®^ Genomic DNA purification kit (USA) according to the manufacturer’s instructions. The concentration (ng/µL) and the purity (A260/A280) of DNA were measured using an ultraviolet NanoDrop spectrophotometer. The accepted purity ratio of the extracted DNA was within 1.8 ± 0.1 [11].

### 2.6. Polymerase Chain Reaction

Specific DNA sequences containing *HMGCR rs17244841*, *APOE rs7412* and *rs429357*, and *SLCO1B1 rs2306283* and *rs11045818* genetic variants were amplified using polymerase chain reaction (PCR). The PCR mixture was prepared in a total volume of 50 µL containing 50 ng of isolated DNA, 10 pmole of each forward and reverse primer (Table S1), and PCR master mixture containing MgCl_2_, dNTPs, Taq DNA polymerase, and free-nuclease water. The primer sequences were obtained from the literature [12,13]. The PCRs were done using a Bio-Rad thermal cycler with the following cycling parameters: initial denaturation for 5 min at 95 °C and 30 cycles of 95 °C for 30 s, 56–62 °C for 30 s, and 72 °C for 30 s. The PCR reaction was completed by a final extension step at 72 °C for 5 min. The PCR products were then separated using 2% agarose gel electrophoresis.

### 2.7. DNA Sequencing

The samples of PCR product were sent to the GENEWIZ Company (South Plainfield, NJ, USA) for sequencing the amplified nucleotide sequences of the targeted *HMGCR*, *APOE*, and *SLCO1B1* genes using the Sanger sequencing method [14]. The same forward and reverse primers used in the PCR reaction were also used for the DNA sequencing. Sequence analysis was performed using an Applied Biosystems Model ABI373x1. The alignment of the DNA sequence was done using Multialign software [15]. The DNA sequence chromatograms were visualized by DNA Based v3.5.4 software (Heracle BioSoft, Romania). The template sequence of the *HMGCR*, *APOE*, and *SLCO1B1* genes in *Homo sapiens* were obtained from the GenBank database [16].

### 2.8. Statistical Analyses

Statistical analyses were performed using the Statistical Package for Social Sciences (SPSS Inc., Chicago, IL, USA) version 23 for Windows. The lipid profile data of the volunteers were normally distributed according to the Kolmogorov–Smirnov test. A one-way analysis of variance (ANOVA) test and Tukey’s HSD post-hoc test were used to analyze the differences in atorvastatin response among different groups of *SLCO1B1 rs2306283* genotypes. The homozygous genotype was not detected among the DM2 patients, for *HMGCR rs17244841, APO rs7412,* and *SLCO1B1 rs11045818* genotypes, so a student t-test was used to compare the mean values between the “wild” and heterozygous genotypes. One-tailed t-test was used to compare between the 2 groups since we hypothesized that these genetic variants decrease atorvastatin response. A difference was considered significant when the *p*-value was less than 0.05. Deviation of the observed genotype frequency from Hardy–Weinberg equilibrium was determined using a chi-squared (X^2^) test.

## 3. Results

### 3.1. Demographics of Patients

The mean age of the patients was 55 ± 6 years, 57% (79 patients) were male, and 43% (60 patients) were female. The average weight of the patients was 86 ± 14.3 kg. Their lipid and glycemic profiles from before and after 3 months of taking atorvastatin are shown in Table 1.

### 3.2. The Frequency of HMGCR rs17244841, APOE rs7412 and rs429357, SLCO1B1 rs2306283 and rs11045818 Genotype among Diabetic Patients

There were 128 volunteers who were genotyped with wild *HMGCR rs17244841A/A,* and 10 carried heterozygous A/T, while only one patient had the homozygous *HMGCR rs17244841 T/T* genotype. The *HMGCR rs17244841 A* allele frequency was 0.96, while the T allele frequency was 0.04. Regarding the *APOE rs7412 T > C* genetic variant, 127 patients carried the wild *T/T* genotype, while 12 patients carried the heterozygous *T/C* genotype. The *APOE rs7412 T* allele frequency was 0.96, while the *A* allele frequency was 0.04.

For the *SLCO1B1 rs2306283 A > G* variant, 33 patients carried the wild *A/A* genotype, 64 carried the *A/G* heterozygous genotype, and 42 carried the *G/G* homozygous genotype. The *SLCO1B1 rs2306283 A* allele frequency was 0.47, while the *G* allele frequency was 0.53. Regarding the *SLCO1B1 rs11045818 G > A* genetic variant, we found that 108 volunteers carried the wild *G/G* genotype, 29 carried the heterozygous *G/A* genotype, and two carried the homozygous *A/A* genotype. The *G* allele frequency was 0.88, while the *A* allele frequency was 0.12. None of the observed genotype frequencies showed a deviation from Hardy–Weinberg equilibrium (*p*-value > 0.05, X^2^).

### 3.3. The Effect of Genetic Variants on Atorvastatin Response

Table 2, Table 3, Table 4 and Table 5 show the association of the genotypes with atorvastatin response. The *HMGCR rs17244841* genotype did not show a significant association (*p*-value > 0.05) with atorvastatin response in terms of lipid profiles, glycemic profiles, and hepatic enzymes (Table 2). The reducing effect of atorvastatin on total cholesterol and LDL blood levels was 3 times higher (*p*-value < 0.05) among wild-type (C/C) carriers than heterozygous *APOE rs7412 C/T* genotype carriers (Table 3).

It is found that plasma cholesterol level decreased by 29% among patients with the wild *SLCO1B1 rs2306283 A/A* genotype, while it decreased by 18.5% among *SLCO1B1 rs2306283 A/T* heterozygous carriers and 14.4% among *SLCO1B1 rs2306283 T/T* homozygous carriers (Table 4). In addition, we found that the ALT level was increased significantly (*p*-value < 0.05) by 9% and 16% among patients with heterozygous *SLCO1B1 rs2306283 A/G* and *SLCO1B1 rs2306283 G/G* genotypes, respectively, while it was not elevated among wild carriers (Table 4). Furthermore, ALT levels were increased significantly (*p*-value < 0.05) by 27% among patients with the heterozygous (*G/A*) genotype than the wild (*G/G*) *SLCO1B1 rs11045818* genotype (Table 5).

### 3.4. SLCO1B1 rs2306283 and rs11045818 Haplotype Frequency and Its Effects on Atorvastatin Response

The frequency of *SLCO1B1 rs2306283* and *rs11045818* haplotypes and its association with atorvastatin response is illustrated in Table 6. The most common haplotype is *SLCO1B1 rs2306283 G* and *rs11045818 G* haplotype, with a frequency of 54%. Additionally, no patient with *SLCO1B1 rs2306283 A* and *rs11045818 A* haplotype was found. We found that *SLCO1B1 rs2306283* and *rs11045818* haplotypes are associated with total cholesterol and ALT blood level alterations induced by atorvastatin treatment (Table 6). Atorvastatin decreased total cholesterol levels more significantly (*p*-value < 0.05) among DM2 patients with *SLCO1B1 rs2306283 A* and *rs11045818 G* haplotype (29%) than patients with *SLCO1B1 rs2306283 G* and *rs11045818 G* (15.5%) haplotype. Furthermore, ALT levels were increased by 27% among patients with *SLCO1B1 rs2306283 G* and *rs11045818 A* haplotype. This elevation is statistically significant (*p*-value < 0.05) in comparison with altered ALT levels among diabetic patients with *SLCO1B1 rs2306283 G* and *rs11045818 G,* and those with *SLCO1B1 rs2306283 G* and *rs11045818 A* haplotypes after atorvastatin therapy.

## 4. Discussion

There is wide inter-individual variation in the atorvastatin response among DM2 patients attending the University of Jordan Hospital. Variation in atorvastatin response can be due to multiple factors, such as patient adherence, health status, and drug–drug interaction [17]. However, we have clearly shown that *APOE rs7412 T* and *SLCO1B1 rs2306283 G* alleles significantly reduced the atorvastatin response, with DM2 patients carrying these alleles having significantly higher lipid levels than patients with wild alleles. Additionally, *SLCO1B1 rs2306283 G* and *rs11045818 A* variants were associated with a significant elevation in ALT levels after atorvastatin treatment. Hence, these variants might play a role in the risk of atorvastatin-induced hepatotoxicity. These findings suggest that *APOE rs7412* and *SLCO1B1 rs2306283* and *rs11045818* genetic variants are potential genetic biomarkers for atorvastatin response among DM2 patients of Jordanian Arabic origin. Further clinical studies are needed to confirm the findings of this study with a larger sample size.

This study is not the first to investigate the influence of genetic variants on atorvastatin response among DM2 patients attending the University of Jordan Hospital. Abdullah et al. (2020) studied the effect of endothelial nitric oxide synthase *rs2070744*, *rs1799983*, and *rs61722009* genetic variants on atorvastatin response among Jordanian DM2 patients [18]. They found that those genetic variants were not associated with atorvastatin response. In addition, Hneet et al. (2020) did not find any influence of *CYP7A1* and *ABCG8* genetic variants on atorvastatin response among DM2 Jordanian patients [19]. However, the present study showed that *APOE rs7412*, *SLCO1B1 rs2306283*, and *rs11045818* variants significantly affected atorvastatin response among DM2 patients of Jordanian Arabic origin for the first time.

Some studies showed that the *HMGCR rs17244841* genetic variant affects statin response [20,21], but we did not find an influence among these patients. This might be due to ethnic differences in the frequency of the *HMGCR rs17244841* variant between Jordanians and other ethnic groups. Additionally, this is the first study to investigate the influence of the *HMGCR rs17244841* variant on statin response among DM2 patients. The diabetic disease causes physiological and biochemical alterations that can mask the effects of genetic variants on drug response [22]. According to the On Pharmacogenomics Knowledge Base (PharmGKB) website, the *HMGCR rs17244841* genetic variant has level 3 clinical evidence, which indicates that not enough clinical results support the clinical implementation of *HMGCR rs17244841* variant in statin therapy [23]. The results of this study support that the *HMGCR rs17244841* variant does not have a significant clinical impact on atorvastatin response among DM2 patients.

ApoE plays a major role as a carrier and transporter of cholesterol in the blood and has a role in the metabolism and elimination of cholesterol. Functional genetic variants in the *APOE* gene affect ApoE function and hence cholesterol transport and statin response [24]. We showed that the *APOE rs7412 T* allele decreased the atorvastatin response, and *APOE rs7412 C/T* genotype DM2 patients had a significantly lower response to atorvastatin. This result is in agreement with the report by Lagos et al. that the *APOE rs7412* variant reduced statin response among Chilean subjects [25]. However, our findings contrast with a previous report that the *APOE rs7412 T* allele increases statin response [26]. Therefore, further clinical studies are needed to find out the exact influence of the *APOE rs7412* variant on atorvastatin response.

According to the ACC/AHA guideline classification of statin intensity [27], 20 mg atorvastatin is effective when it produces a 30 to 50% decrease in LDL and blood lipid levels. We found that atorvastatin decreased TC and LDL by 22 and 31%, respectively, among patients with wild *APO rs7412* genotype. Besides, atorvastatin decreased TC and LDL by only 7.5% and 10%, respectively, among patients with heterozygous *APO rs7412* genotype. Accordingly, DM2 patients with wild *APO rs7412* genotype responded, while patients with heterozygous genotype did not respond, to atorvastatin therapy. Therefore, DM2 patients with heterozygous *APO rs7412* genotype are at higher risk of treatment failure with atorvastatin, which may lead to cardiovascular complications.

The major site of action of statins is the liver. Statins enter hepatic cells through SLCO1B1. It is reported that the *SLCO1B1 rs2306283 G* variant decreases the capacity of SLCO1B1 transporter [28]. Accordingly, the influx of statins inside hepatic cells is reduced in patients with the *SLCO1B1 rs2306283 G* allele, and the anti-hypercholesterolemic effect is decreased. This could explain the reduced atorvastatin response among DM2 patients with the *SLCO1B1 rs2306283 G* genotype.

Some studies showed that *SLCO1B1* genetic variants are strongly associated with statin-induced myopathy [29]. We found that DM2 patients with *SLCO1B1 rs2306283 G* and *rs11045818 A* alleles have a significantly higher blood level of ALT enzyme, although the mean of the elevated ALT level was within the normal ALT range (<30 IU/L). Elevated ALT levels might indicate a higher risk of hepatotoxicity [30]. It was reported that *SLCO1B1* genetic variants are associated with drug-induced hepatotoxicity [31,32].

The mechanism of how *SLCO1B1 rs2306283 G* and *rs11045818 A* variants can affect ALT level is still not clear. Although *SLCO1B1 rs11045818 A* is a synonymous variant, it might be in a linkage disequilibrium with other functional variants that have a clinical influence on atorvastatin response. Using Haploview software [33], we found a moderate LD (D = 0.8) between *SLCO1B1 rs11045818* and the functionally non-synonymous *SLCO1B1 rs2306283*. The level of clinical evidence of *SLCO1B1 rs2306283* and *rs11045818* on the PharmGKB website is 3. Since we found a significant association between these variants and atorvastatin response, we recommend investigating these variants’ effects on atorvastatin response among different ethnic groups with a larger sample size.

There are some limitations to this study. First, the sample size was relatively small, and these findings should be confirmed using a larger sample size. Second, other factors affecting statin response, such as drug–drug interactions, drug–food interactions, and patient adherence, were not included in this study. Third, genetic variants of cytochrome P450s, which metabolize statins, were not studied. Lastly, the patients were on adjusted doses of anti-diabetic metformin therapy, which affects the lipid and glycemic profiles of DM2 patients.

## 5. Conclusions

In conclusion, *APOE rs7412, SLCO1B1 rs2306283,* and *rs11045818* genotypes have an influence on atorvastatin response. Therefore, they can be considered as potential genetic biomarkers of atorvastatin therapy among DM2 patients of Jordanian Arabic origin. Further clinical studies with larger sample numbers are needed to confirm these findings.

## Figures and Tables

**Table 1:** Lipid, glycemic, and hepatic enzyme levels in the blood of diabetic patients before and after 3 months of atorvastatin treatment.

	Lipid, Glycemic and Hepatic Enzyme in the Blood							
**Time of Recording**	TC (mg/dl)	LDL (mg/dl)	HDL (mg/dl)	TGs (mg/dl)	ALT (IU/L)	AST (IU/L)	HbA1c	FBG
**Before Atorvastatin Treatment**	190.42 ± 46.94	133.59 ± 42.71	44.73 ± 11.58	183.81 ± 91.58	18.05 ± 6.60	21.37 ± 10.57	8.62 ± 1.88	221 ± 52
**After 3 Months of Atorvastatin Treatment**	151.16 ± 30.98 *	93.54 ± 28.81 *	43.09 ± 14.85	165.07 ± 74.05	19.27 ± 7.96	22.37 ± 11.60	7.57 ± 1.45 *	182 ± 47 *

Abbreviations: TC: total cholesterol; LDL: low-density lipoprotein; HDL: high-density lipoprotein; TGs: triglyceride; HbA1c: glycated hemoglobin; ALT: alanine aminotransferase; AST: aspartate transaminase; FBG: fasting blood glucose. Values shown are means ± SD. “*” indicates statistical difference with *p*-value < 0.05, paired *t*-test.

**Table 2:** The association of *HMGCR rs17244841* genotype with atorvastatin response among diabetic Jordanian patients.

*HMGCR RS17244841* GENOTYPE	Δ TC (MG/DL)%	Δ LDL (MG/DL)%	Δ HDL (MG/DL)%	Δ TGS (MG/DL)%	Δ ALT (IU/L)%	Δ AST (IU/L)%	Δ HbA1c
***A/A***	−19.81 ± 18.68	−28.17 ± 22.53	1.48 ± 4.02	−4.08 ± 41.44	6.13 ± 36.74	−1.61 ± 39.75	−11.88 ± 9.464
***A/T***	−27.66 ± 11.71	−39.49 ± 15.67	−9.76 ± 22.78	−7.69 ± 53.03	−13.94 ± 28.65	−3.93 ± 36.22	13.98 ± 29.24
***P* VALUE**	0.53	0.39	0.85	0.58	0.36	0.91	0.06

Abbreviations: TC: total cholesterol; LDL: low-density lipoprotein; HDL: high-density lipoprotein; TGs: triglyceride; HbA1c: glycated hemoglobin; ALT: alanine aminotransferase; AST: aspartate transaminase. Values shown are means ± SD. A negative value indicates a decrease, while a positive value indicates an increase, in the blood level of the lipid, glycemic, and hepatic enzyme after 3 months of atorvastatin treatment. The results of homozygous T/T patients were excluded in the statistical comparison, since their frequency is less than 3. Independent one-tail *t*-test was used to compare the values of wild and heterozygous genotypes.

**Table 3:** The association of *APO rs7412* genotype with atorvastatin response among diabetic Jordanian patients.

*APO RS7412* GENOTYPE	Δ TC (MG/DL)%	Δ LDL (MG/DL)%	Δ HDL (MG/DL)%	Δ TGS (MG/DL)%	Δ ALT (IU/L)%	Δ AST (IU/L)%	Δ HbA1
***C/C***	−21.85 ± 18.18	−30.98 ± 21.76	1.43 ± 54.6	−5.92 ± 42.72	5.92 ± 42.74	−0.39 ± 36.1	−10.37 ± 21.99
***C/T***	−7.57 ± 13.16	−10.22 ± 17.49	−6.08 ± 15.27	4.42 ± 37.52	−3.07 ± 48.60	−14.17 ± 64.56	−7.37 ± 11.98
***P* VALUE**	0.03 *	0.01 *	0.68	0.49	0.61	0.42	0.67

Abbreviations: TC: total cholesterol; LDL: low-density lipoprotein; HDL: high-density lipoprotein; TGs: triglyceride; HbA1c: glycated hemoglobin; ALT: alanine aminotransferase; AST: aspartate transaminase. Values shown are % mean ± SD. A negative value indicates a decrease, while a positive value indicates an increase, in the blood level of the lipid, glycemic, and hepatic enzyme after 3 months of atorvastatin treatment. “*” Indicates statistical significance (*p*-value < 0.05, *t*-test).

**Table 4:** The association of *SLCO1B1 rs2306283* genotype with atorvastatin response among diabetic Jordanian patients.

*SLCO1B1 RS2306283* GENOTYPE	Δ TC (MG/DL)%	Δ LDL (MG/DL)%	Δ HDL (MG/DL)%	Δ TGS (MG/DL)%	Δ ALT (IU/L)%	Δ AST (IU/L)%	Δ HbA1c
***A/A***	−28.81 ± 13.06	−37.02 ± 17.28	−5.18 ± 13.04	−17.14 ± 38.61	−13.43 ± 26.81	−16.30 ± 28.71	−15.24 ± 22.23
***A/G***	−18.49 ± 21.18	−27.62 ± 25.38	−6.75 ± 15.31	3.33 ± 41.7	9.27 ± 39.9	4.74 ± 36.9	−6.84 ± 21.54
***G/G***	−14.39 ± 18.19	−28.96 ± 22.11	0.67 ± 51.9	−4.87 ± 42.17	15.97 ± 36.41	−1.77 ± 39.24	−1.77 ± 39.24
***P* VALUE**	0.04 *	0.12	0.17	0.19	0.02 *	0.27	0.30

Abbreviations: TC: total cholesterol; LDL: low-density lipoprotein; HDL: high-density lipoprotein; TGs: triglyceride; HbA1c: glycated hemoglobin; ALT: alanine aminotransferase; AST: aspartate transaminase. Values shown are means ± SD. A negative value indicates a decrease, while a positive value indicates an increase, in the blood level of the lipid, glycemic, and hepatic enzyme after 3 months of atorvastatin treatment. “*” Indicates statistical significance (*p*-value < 0.05, ANOVA).

**Table 5:** The association of *SLCO1B1 rs11045818* genotype with atorvastatin response among diabetic Jordanian patients.

*SLCO1B1 RS11045818* GENOTYPE	Δ TC (MG/DL)%	Δ LDL (MG/DL)%	Δ HDL (MG/DL)%	Δ TGS (MG/DL)%	Δ ALT (IU/L)%	Δ AST (IU/L)%	Δ HbA1c
***G/G***	−19.86 ± 19.28	−28.29 ± 23.12	−5.80 ± 15.53	−2.81 ± 42.98	0.94 ± 36.67	−2.29 ± 33.83	−2.29 ± 33.83
***G/A***	−22.07 ± 14.48	−31.21 ± 18.61	9.6 ± 22.3	−11.43 ± 39.62	27.14 ± 27.05	0.11 ± 56.4	−9.08 ± 18.40
***P* VALUE**	0.63	0.61	0.32	0.41	0.01 *	0.85	0.84

Abbreviations: TC: total cholesterol; LDL: low-density lipoprotein; HDL: high-density lipoprotein; TGs: triglyceride; HbA1c: glycated hemoglobin; ALT: alanine aminotransferase; AST: aspartate transaminase. Values shown are means ± SD. A negative value indicates a decrease, while a positive value indicates an increase, in the blood level of the lipid, glycemic, and hepatic enzyme after 3 months of atorvastatin treatment. “*” Indicates statistical significance (*p*-value < 0.05, *t*-test). The results of homozygous A/A patients were excluded in the statistical comparison, since their frequency is less than 3.

**Table 6:** The association of *SLCO1B1 rs2306283* and *rs11045818* haplotype with atorvastatin response among diabetic Jordanian patients.

SLCO1B1 HAPLOTYPE		N (%)	Δ TC (MG/DL)%	Δ LDL (MG/DL)%	Δ HDL (MG/DL)%	Δ TGS (MG/DL)%	Δ ALT (IU/L)%	Δ AST (IU/L)%	Δ HbA1c
**rs2306283**	**rs11045818**								
**A**	**G**	33 (23.7)	−28.81 ± 13.83	−37.02 ± 17.28	−5.18 ± 13.04	−17.41 ± 38.61	−13.43 ± 26.82	−16.30 ± 28.71	−15.24 ± 22.69
**G**	**G**	75 (54)	−15.48 ± 17.41	−24.12 ± 24.52	−6.09 ± 16.72	4.20 ± 43.66	6.32 ± 38.73	3.07 ± 34.5	−7.90 ± 21.63
**G**	**A**	31 (22.3)	−22.07 ± 14.17	−29.21 ± 18.61	11.62 ± 62.3	−11.43 ± 39.63	27.14 ± 27.05	0.11 ± 56.38	−9.65 ± 18.16
**P VALUE**			0.02 *	0.07	0.13	0.41	0.04 *	0.32	0.39

Abbreviations: TC: total cholesterol; LDL: low-density lipoprotein; HDL: high-density lipoprotein; TGs: triglyceride; HbA1c: glycated hemoglobin; ALT: alanine aminotransferase; AST: aspartate transaminase. Values shown are means ± SD. A negative value indicates a decrease, while a positive value indicates an increase, in the blood level of the lipid, glycemic, and hepatic enzyme after 3 months of atorvastatin treatment. “*” Indicates statistical significance (*p*-value < 0.05, ANOVA).

## Supplementary Materials

The following are available online at https://www.mdpi.com/2075-1729/10/10/232/s1, Table S1: title Name and sequence of oligonucleotide primers used in polymerase chain reaction of *HMGCR*, *APOE*, and *SLCO1B1* genes.
