# Supplementary material for: The Association of 3-Hydroxy-3-Methylglutaryl-CoA Reductase, Apolipoprotein E, and Solute Carrier Organic Anion Genetic Variants with Atorvastatin Response among Jordanian Patients with Type 2 Diabetes
Source: Life (Basel). 2020 Oct 5;10(10):232. doi: 10.3390/life10100232 (PMC7599896; doi:10.3390/life10100232)
Supplement: Supplementary file 1 [file life-10-00232-s001.pdf]

Table 1. Name and sequence of oligonucleotide primers used in polymerase chain reaction of *HMGCR*, *APOE*, and *SLCO1B1* genes.

Name	Sequence
HMGCR rs17244841 (F)	5'- CAGGCATAGAGTCCACAAGC -3'
HMGCR rs17244841 (R)	5'- TGGACACAATGGATTAGGCTG -3'
APOE rs7412 and *rs429357* (F)	5'-GCACGGCTGTCCAAGGAGCTGC-3'
APOE rs7412 and *rs429357* (R)	5'-GGCGCTCGCGGATGGCGCTGAG-3'
SLCO1B1 rs2306283 and *rs11045818* (F)	5'-GCAAATAAAGGGGAATATTTCTC-3'
SLCO1B1 rs2306283 and *rs11045818* (R)	5'-AGAGATGTAATTAAATGTATAC-3'
